# Efficacy of Histaglobulin in Allergic Diseases: A Systematic Review and Meta-Analysis with Implications for Allergic Rhinitis

**DOI:** 10.3390/ijms27177753

**Published:** 2026-08-29

**Authors:** Jayoung Oh, Ji Ho Choi

**Affiliations:** 1 Department of Otorhinolaryngology–Head and Neck Surgery, Soonchunhyang University Cheonan Hospital, Soonchunhyang University College of Medicine, Cheonan 31151, Republic of Korea; planet90@gmail.com; 2 Department of Otorhinolaryngology–Head and Neck Surgery, Soonchunhyang University Bucheon Hospital, Soonchunhyang University College of Medicine, Bucheon 14584, Republic of Korea

**Keywords:** histaglobulin, histamine–immunoglobulin complex, Histobulin, allergic rhinitis, chronic urticaria, immunotherapy, systematic review, meta-analysis

## Abstract

Histaglobulin (histamine/immunoglobulin complex) has been used for decades as a non-allergen-specific immunotherapy for allergic diseases including allergic rhinitis, chronic urticaria, and atopic dermatitis. Despite growing clinical interest, no meta-analysis has synthesized the available evidence. This systematic review and meta-analysis aimed to evaluate the efficacy and safety of histaglobulin across allergic diseases, with particular emphasis on implications for allergic rhinitis management. PubMed, Embase, Web of Science, and Cochrane Library were searched from inception to March 2026. Studies evaluating subcutaneous histaglobulin in patients with allergic diseases were included. For chronic urticaria studies reporting Urticaria Activity Score (UAS) as mean ± standard deviation, a random-effects meta-analysis was performed using the standardized mean difference (Hedges’ g). Allergic rhinitis studies were synthesized narratively. Risk of bias was assessed using the National Institutes of Health (NIH) Quality Assessment Tools. The protocol was registered in the International Prospective Register of Systematic Reviews (PROSPERO). Fourteen studies (N = 754) met the inclusion criteria: six chronic urticaria studies with quantitative data (N = 190), three allergic rhinitis studies (N = 165), two atopic dermatitis/chronic spontaneous urticaria case series (N = 8), and three chronic urticaria studies with incomplete data. Meta-analysis of six chronic urticaria studies, all of which used an uncontrolled before–after design, demonstrated a large pooled within-group effect size (Hedges’ g = 2.82; 95% confidence interval [CI] 1.75–3.88; *p* < 0.00001), with high heterogeneity (I^2^ = 98.2%). UAS reduction ranged from 44% to 89% across studies. Complete remission rates ranged from 30% to 89%. Sensitivity analysis excluding one study with reporting concerns yielded consistent results (g = 2.34; 95% CI 1.83–2.85; I^2^ = 90.2%). All three allergic rhinitis studies reported significant symptom improvement (response rates 57–90%) with normalization of serum immunoglobulin E (IgE) and eosinophil counts. No serious adverse events were identified within the available literature; however, the cumulative sample size (N = 754) remains insufficient to exclude rare or long-term adverse effects. Available evidence suggests that histaglobulin may reduce disease activity in chronic urticaria; because the pooled estimate derives predominantly from uncontrolled before–after studies with substantial heterogeneity, it reflects within-group improvement following treatment rather than a controlled estimate of treatment efficacy, and confidence in the effect remains limited. Preliminary evidence suggests that histaglobulin may also provide benefit in patients with allergic rhinitis; however, the available data are limited and heterogeneous, and larger, well-designed controlled studies are needed to confirm its effectiveness. Well-designed randomized controlled trials are needed, particularly for allergic rhinitis, to establish histaglobulin’s role in evidence-based otorhinolaryngology practice.

## 1. Introduction

Allergic diseases represent a growing global health burden, with allergic rhinitis alone affecting 10–30% of the world population and chronic urticaria impacting 0.5–5% of the general population [1,2]. While second-generation antihistamines remain the first-line treatment for both conditions, a substantial proportion of patients remain inadequately controlled, necessitating stepped-up therapy including immunomodulators, biologics such as omalizumab, or allergen-specific immunotherapy [3,4]. However, these advanced therapies are limited by high cost, potential adverse effects, or the requirement for specific allergen identification.

Histaglobulin (also known as Histobulin™, Histoglob^®^, or histaglobin) is a non-allergen-specific immunotherapeutic preparation composed of human normal immunoglobulin (12 mg) complexed with histamine dihydrochloride (0.15 μg) in strictly defined proportions [5]. Originally developed by Parrot and Laborde in the 1950s for restoring histaminopexy (the serum’s ability to bind and inactivate histamine), histaglobulin has been approved for allergic rhinitis, bronchial asthma, and chronic urticaria in several countries including Korea, India, Japan, and Russia [6,7].

The proposed mechanisms of action are multifaceted. Histaglobulin induces the production of anti-histamine antibodies that neutralize endogenously released histamine, thereby increasing serum histamine-binding capacity from 0–5% in allergic patients toward the normal range of 20–30% [8]. Additionally, histaglobulin inhibits antigen-induced histamine release from mast cells and basophils [9], suppresses allergen-induced eosinophil accumulation without affecting neutrophils [10], inhibits NF-κB nuclear translocation [11], and modulates the T helper type 1 (Th1)/T helper type 2 (Th2) balance [12]. Recent evidence suggests it may also decrease serum total immunoglobulin E (IgE) levels [13].

Despite its decades-long clinical use and approved indications, histaglobulin remains largely unrecognized in Western allergy and otorhinolaryngology guidelines. Clinical evidence has been scattered across regional journals in India, Korea, Russia, and Japan, with no prior meta-analysis synthesizing the available data. From an otorhinolaryngology perspective, allergic rhinitis is one of the approved indications for histaglobulin, yet the evidence base specifically for this indication remains poorly characterized.

This systematic review and meta-analysis aims to (1) comprehensively evaluate the efficacy and safety of histaglobulin across all allergic diseases studied to date; (2) perform quantitative pooling where data permit; and (3) specifically examine the evidence and implications for allergic rhinitis management, thereby informing future research priorities in otorhinolaryngology.

## 2. Materials and Methods

This systematic review and meta-analysis was conducted in accordance with the Preferred Reporting Items for Systematic Reviews and Meta-Analyses (PRISMA) 2020 guidelines [14] and the Cochrane Handbook for Systematic Reviews of Interventions [15]. The protocol was prospectively registered in PROSPERO (CRD420261381448).

### 2.1. Search Strategy

Four electronic databases (PubMed/MEDLINE, Embase, Web of Science, and Cochrane Library) were searched from inception to March 2026 without date restrictions. The search strategy combined terms for the intervention (“histaglobulin” OR “histobulin” OR “histaglobin” OR “histamine immunoglobulin” OR “histamine gamma globulin” OR “histamine-fixed immunoglobulin”) with terms for allergic diseases (“allergic rhinitis” OR “urticaria” OR “atopic dermatitis” OR “asthma” OR “allergy”). The full search strategies for each database are provided in Supplementary Table S1. Additionally, ClinicalTrials.gov, WHO ICTRP, reference lists of included studies, and Google Scholar were searched for additional eligible studies. The search was restricted to English-language publications.

### 2.2. Eligibility Criteria

Studies were included if they: (1) enrolled patients with any allergic disease (allergic rhinitis, chronic urticaria, atopic dermatitis, or bronchial asthma); (2) administered subcutaneous histaglobulin (histamine/immunoglobulin complex) as the primary intervention; (3) reported clinical outcomes using validated scoring systems or response rates; and (4) were original clinical studies (interventional or observational). Exclusion criteria comprised: non-English publications, conference abstracts without full text, narrative reviews, purely preclinical studies (except for mechanistic context), studies using histaglobulin solely as a combination with another experimental intervention, and duplicate publications.

### 2.3. Study Selection and Data Extraction

Two reviewers independently screened titles, abstracts, and full texts. Disagreements were resolved by consensus. Data were extracted using a standardized form including study design, sample size, patient demographics, disease type, intervention protocol (dose, frequency, duration), outcome measures (UAS, symptom scores, IgE levels, eosinophil counts), treatment response rates, adverse events, and follow-up duration.

### 2.4. Risk of Bias Assessment

Risk of bias was assessed using the National Institutes of Health (NIH) Quality Assessment Tools appropriate to each study design: the NIH tool for controlled intervention studies (for RCTs), the NIH tool for before-after studies with no control group (for single-arm prospective studies), and the NIH tool for case series [16]. Each study was rated as “good,” “fair,” or “poor” quality. The overall certainty of evidence was assessed using the Grading of Recommendations, Assessment, Development, and Evaluations (GRADE) framework [17].

### 2.5. Statistical Analysis

For chronic urticaria studies reporting pre- and post-treatment UAS as mean ± standard deviation (SD), the standardized mean difference (SMD) was calculated using Hedges’ g with correction for small sample bias. A pre–post correlation coefficient of r = 0.5 was assumed, with sensitivity analyses conducted at r = 0.3, 0.7, and 0.8. Heterogeneity was assessed using Cochran’s Q test and the I^2^ statistic, with I^2^ > 75% indicating substantial heterogeneity. A random-effects model using the DerSimonian–Laird method was employed given the expected clinical and methodological heterogeneity. Leave-one-out sensitivity analysis was performed. Because the great majority of eligible studies were uncontrolled before–after studies, the pooled estimate was interpreted as representing within-group change from baseline rather than a between-group (placebo-controlled) estimate of treatment efficacy. Potential sources of heterogeneity were explored descriptively according to the Urticaria Activity Score (UAS) instrument used (daily vs. weekly), treatment duration and number of injections, baseline disease severity, study design (single-arm vs. comparator-controlled), study type (prospective clinical trial vs. post-marketing surveillance or retrospective real-world study), publication period, and geographical setting; formal subgroup analyses and meta-regression were not undertaken because the number of pooled studies (k = 6) was below the minimum generally recommended for such analyses [15]. Publication bias was assessed visually using funnel plots. For allergic rhinitis studies lacking continuous outcome data with SD, narrative synthesis was performed following the Synthesis Without Meta-analysis (SWiM) guidelines [18]. Statistical analyses were performed using Python 3.11 (Python Software Foundation, Wilmington, DE, USA) with the SciPy 1.11.4 and NumPy 1.26.2 libraries.

## 3. Results and Discussion

### 3.1. Study Selection

**Figure 1:**
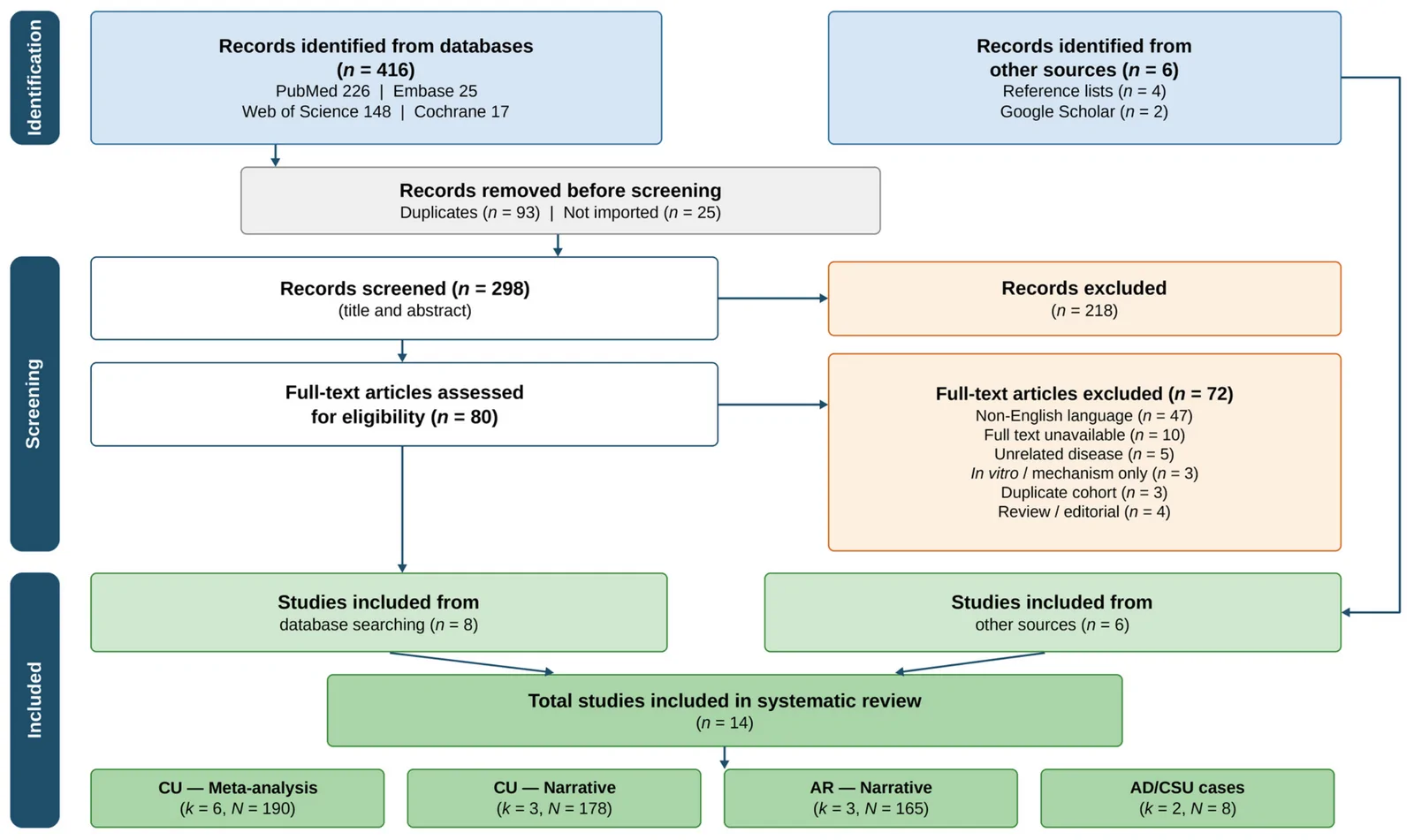
PRISMA 2020 flow diagram of study selection. PRISMA, Preferred Reporting Items for Systematic Reviews and Meta-Analyses; AD, atopic dermatitis; AR, allergic rhinitis; CSU, chronic spontaneous urticaria; CU, chronic urticaria; k, number of studies; N, number of participants; n, number of records.

The database search across four databases yielded 416 records (PubMed 226, Embase 25, Web of Science 148, Cochrane Library 17). After importing 391 records into Rayyan (Rayyan Systems Inc., Cambridge, MA, USA) and removing 93 duplicates, 298 records were screened by title and abstract, of which 80 full-text articles were assessed for eligibility. Seventy-two articles were excluded at full-text review (47 non-English language, 10 full text unavailable, 5 unrelated disease, 3 in vitro/mechanism only, 3 duplicate cohort, 4 review/editorial). Eight studies were identified through database searching, and an additional six studies were identified through reference list checking (*n* = 4) and Google Scholar (*n* = 2). In total, 14 studies met the final inclusion criteria (Figure 1).

### 3.2. Study Characteristics

The 14 included studies comprised: nine chronic urticaria studies, three allergic rhinitis studies, and two atopic dermatitis/chronic spontaneous urticaria case series. The studies were published between 1997 and 2025, with the majority (*n* = 12) from India and two from Korea. Total enrollment across clinical studies was 754 patients. All studies used subcutaneous histaglobulin at the standard composition of 12 mg human IgG/0.15 μg histamine dihydrochloride per 1–2 mL injection. The twelve Indian studies used Histoglob^®^ (Bharat Serums and Vaccines Ltd., Ambernath, India) [5], whereas the two Korean case series used Histobulin™ (Green Cross PD Co., Ochang, Republic of Korea) [7]. Both preparations are histamine-fixed human normal immunoglobulins with the same declared composition and are subject to lot-release testing of histamine content [7], so that formulation-related differences between studies are expected to be minimal; nevertheless, no head-to-head comparison of the two products has been published. Treatment protocols varied from 3 weekly injections to 46 weekly injections, with the most common protocol being 8 weekly injections (Table 1).

### 3.3. Chronic Urticaria: Meta-Analysis

**Figure 2:**
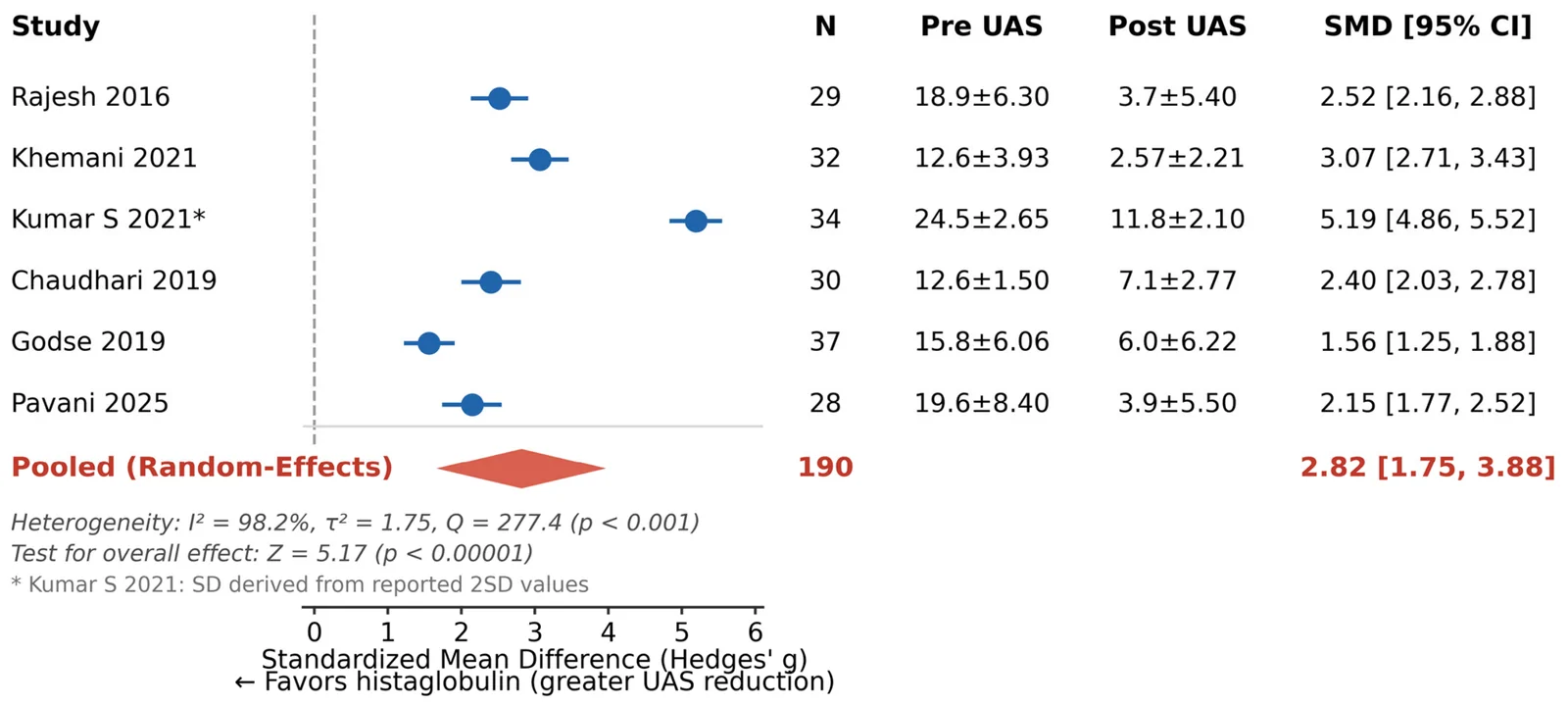
Forest plot of the meta-analysis of histaglobulin efficacy in chronic urticaria (Pre-Post UAS Change, Random-Effects Meta-Analysis, k = 6, N = 190). The pooled standardized mean difference (Hedges’ g) was calculated using a random-effects model (DerSimonian–Laird). The six pooled studies are Rajesh 2016 [19], Khemani 2021 [20], Kumar S 2021 [21], Chaudhari 2019 [22], Godse 2019 [23], and Pavani 2025 [24]. Blue circles represent the point estimate of each individual study and the horizontal bars the corresponding 95% CI; the diamond represents the pooled effect estimate. The leftward arrow below the horizontal axis indicates the direction of effect favouring histaglobulin, that is, a greater reduction in UAS. * Kumar S 2021 reported dispersion as mean ± 2SD; the SD was derived by dividing by 2. CI, confidence interval; SD, standard deviation; SMD, standardized mean difference; UAS, Urticaria Activity Score.

Six chronic urticaria studies (N = 190) [19,20,21,22,23,24] provided sufficient quantitative data (pre- and post-treatment UAS as mean ± SD) for meta-analytic pooling (Table 2). The pooled effect size using a random-effects model was large and statistically significant (Hedges’ g = 2.82; 95% CI 1.75–3.88; Z = 5.17; *p* < 0.00001), indicating a substantial reduction in disease activity following histaglobulin treatment (Figure 2). Because all six pooled studies were uncontrolled before–after cohorts, this estimate quantifies within-group change from baseline and cannot be equated with a placebo-controlled treatment effect. Heterogeneity was very high (I^2^ = 98.2%; Q = 277.4; *p* < 0.001; τ^2^ = 1.75), indicating that the pooled estimate should be regarded as an average across clinically and methodologically disparate studies rather than as a single common effect. Several sources of inconsistency can be identified. First, the outcome instrument differed: four studies used a daily UAS with a maximum of 18–33 points [19,21,22,24], whereas two used the weekly UAS7 (maximum 42 points) [20,23]; differences in scale range, ceiling, and recall period directly affect both the mean change and its dispersion, and hence the standardized effect size. Second, treatment intensity and duration varied more than two-fold, from three injections over 3 weeks [23] through six weekly injections [21] to eight weekly injections [19,20,22,24], and the study with the shortest exposure yielded the smallest effect (g = 1.56). Third, baseline disease severity ranged widely (mean pre-treatment UAS 12.6–24.5); the three studies with the highest baseline scores reported effect sizes of g = 2.15–5.19, compared with g = 1.56–3.07 in the three studies with lower baseline scores, a gradient compatible with a contribution from regression to the mean. Fourth, study design and study type differed: four studies were single-arm before–after cohorts [19,20,23,24] whereas two were comparator-controlled studies of histaglobulin versus autologous serum therapy [21,22], and one was a post-marketing surveillance study [23] rather than a prospective clinical trial, with correspondingly different patient selection, monitoring intensity, and permitted co-interventions. Fifth, the study contributing the largest effect (g = 5.19) reported dispersion as mean ± 2SD, requiring derivation of the standard deviation; its exclusion reduced I^2^ from 98.2% to 90.2% (Section 3.4), identifying it as the single largest contributor to inconsistency. Sixth, the pooled studies span nearly a decade (2016–2025) and, although all six were conducted in India, they differed in clinical setting (single-centre tertiary dermatology practice vs. multi-centre post-marketing surveillance) and in the concomitant antihistamine use permitted during follow-up. Taken together, however, these factors do not fully account for an I^2^ of 98.2%, and a substantial proportion of the observed inconsistency remains unexplained; the pooled estimate should therefore be regarded as hypothesis-generating rather than as a precise summary of a common treatment effect.

Across the six studies, the mean UAS reduction ranged from 43.7% to 89.2%. Complete remission rates (UAS = 0) were reported in five studies [19,20,23,24,27] and ranged from 30% to 89.2% at 8–24 weeks follow-up. The mean antihistamine pill burden was significantly reduced in all studies reporting this outcome. Pre- and post-treatment UAS values across studies are compared in Supplementary Figure S1.

### 3.4. Sensitivity Analyses

Leave-one-out analysis demonstrated that the pooled effect remained large and significant regardless of which study was excluded (range: g = 2.34–3.07). Notably, excluding Kumar S 2021 [21] (which reported mean ± 2SD rather than mean ± SD, requiring SD derivation) reduced heterogeneity substantially (g = 2.34; 95% CI 1.83–2.85; I^2^ = 90.2%). Sensitivity analysis for the assumed pre-post correlation coefficient showed stable results across r = 0.3 to r = 0.8, with pooled g consistently at 2.82 and 95% CIs ranging from [1.70, 3.93] to [1.76, 3.87] (Table 3).

### 3.5. Publication Bias

**Figure 3:**
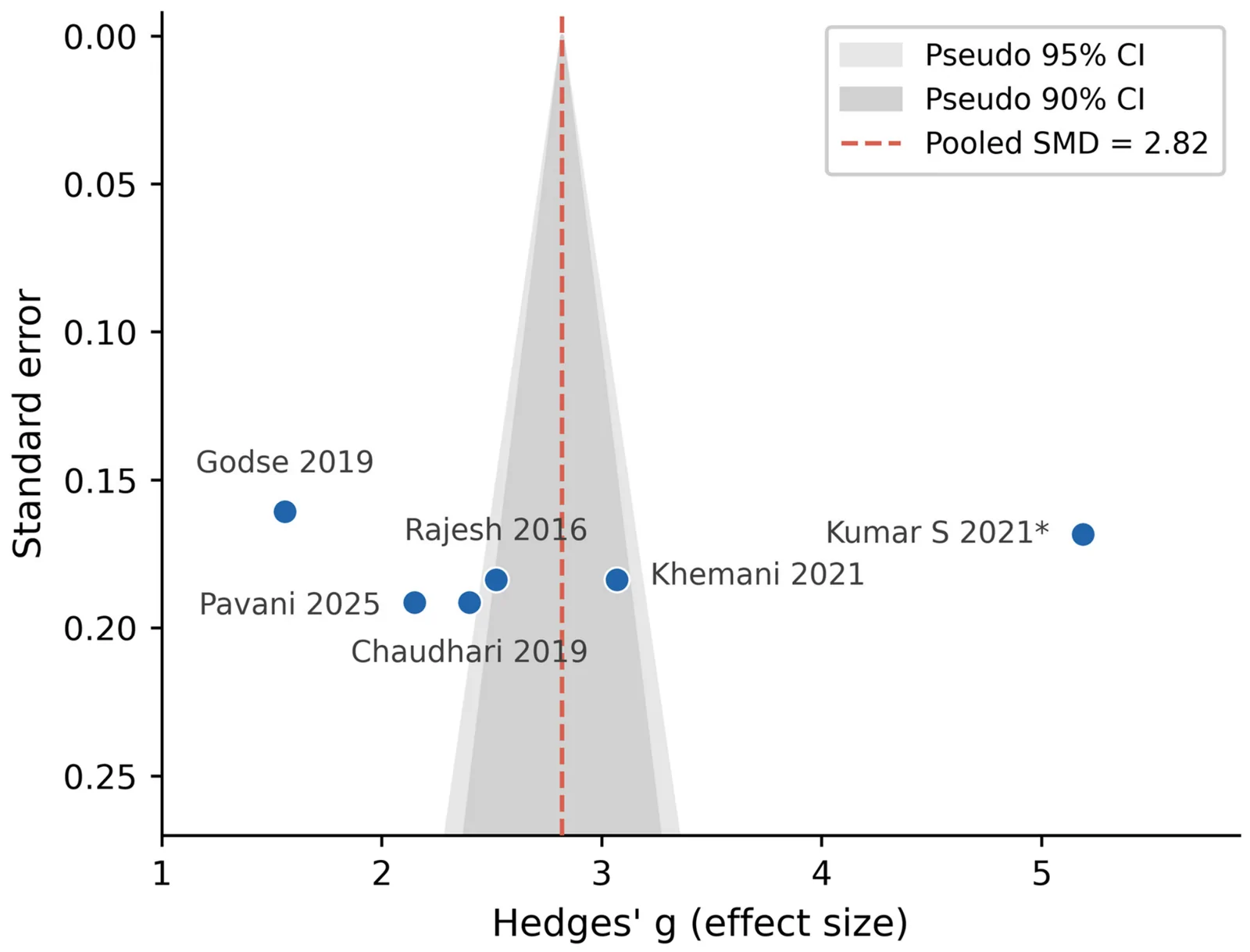
Funnel plot for visual assessment of publication bias. The dashed line represents the pooled effect estimate. The light and dark shaded regions represent the pseudo 95% and pseudo 90% confidence intervals, respectively. Each blue circle represents one included study: Rajesh 2016 [19], Khemani 2021 [20], Kumar S 2021 [21], Chaudhari 2019 [22], Godse 2019 [23], and Pavani 2025 [24]. * Kumar S 2021 reported dispersion as mean ± 2SD; the SD was derived by dividing by 2. CI, confidence interval; SD, standard deviation; SE, standard error; SMD, standardized mean difference.

Visual inspection of the funnel plot (Figure 3) showed moderate asymmetry, with smaller studies tending to report larger effect sizes. However, given only six studies, formal statistical tests for funnel plot asymmetry were not performed, as these tests have inadequate power when k < 10 [15].

### 3.6. Chronic Urticaria: Additional Studies

Three additional chronic urticaria studies could not be included in the quantitative meta-analysis due to incomplete reporting of SD values or use of non-continuous outcome measures. Kaur et al. [25] reported a 73.6% reduction in mean UAS (20.1 to 5.33) in 28 patients. Ravi et al. [26] demonstrated improvement in all four treatment subgroups (autologous serum skin test [ASST]-positive and ASST-negative patients treated with either autologous serum therapy [AST] or histaglobulin), with histaglobulin showing particular benefit in ASST-negative patients (UAS 15.6 to 1.65, *p* = 0.002). Kumar YH et al. [27] reported that 81.25% of ASST-positive patients and 43.54% of ASST-negative patients improved with histaglobulin combined with short-course oral prednisolone.

### 3.7. Allergic Rhinitis

Three studies (N = 165) evaluated histaglobulin specifically in allergic rhinitis. None provided continuous outcome data suitable for meta-analytic pooling; therefore, narrative synthesis was performed.

Narayana et al. [28] conducted the earliest English-language study in 54 patients with allergic rhinitis, comparing three arms: histaglobulin alone (*n* = 18), histaglobulin plus terfenadine (*n* = 18), and histaglobulin plus cetirizine (*n* = 18). The overall clinical response rate at 10 weeks was 59.6% for histaglobulin alone, 61.7% for histaglobulin plus terfenadine, and 56.9% for histaglobulin plus cetirizine, with no significant advantage of combination therapy. At 6-month follow-up, the recurrence rate was low (2/13 in the histaglobulin alone group). However, blood eosinophil counts and serum IgE levels did not show statistically significant changes.

Verma et al. [29] studied 50 allergic rhinitis patients unresponsive to conventional therapy, using a different dosing protocol (4 injections at 4-day intervals for two consecutive months, followed by monthly injections for 3 months and a booster at 6 months). The overall response rate was 88%, with significant improvement in all symptom parameters (nasal obstruction, rhinorrhea, sneezing, and itching; all *p* < 0.05). Serum IgE levels normalized in 94% of patients, and absolute eosinophil counts normalized in 84%. Only 6/50 patients showed poor response or recurrence at 1-year follow-up.

Manohar and Selvakumaran [13] studied 61 patients with allergic asthma and/or allergic rhinitis, measuring serum IgE before and after histaglobulin treatment. IgE reduction was observed in 90.2% (55/61) of patients, with 5 patients achieving >80% reduction and 11 achieving 50–80% reduction in serum IgE levels. Detailed results of the allergic rhinitis studies are presented in Supplementary Table S2. These three studies differed substantially in design, dosing protocol, outcome definition, and endpoint, and none included an untreated, placebo, or active comparator arm, so that quantitative pooling was neither feasible nor appropriate. Preliminary evidence therefore suggests that histaglobulin may provide benefit in patients with allergic rhinitis; however, the available data are limited and heterogeneous, and larger, well-designed controlled studies are needed to confirm its effectiveness.

### 3.8. Atopic Dermatitis and Chronic Spontaneous Urticaria

Two case series by Noh and colleagues [30,31] reported the use of Histobulin™ in atopic dermatitis (4 cases, weekly subcutaneous injections for 24–36 weeks) and chronic spontaneous urticaria (4 cases, weekly injections continued until remission). In the atopic dermatitis series, the Scoring Atopic Dermatitis (SCORAD) index improved from a mean of 32.9 to 8.9 points, with one patient achieving complete remission (SCORAD 42.5 → 0 after 36 injections). All four patients showed reduced frequency of upper respiratory infections during treatment. In the CSU series, all four patients achieved sustained remission (10–52 months without recurrence), with two early responders (12 injections) and two late responders (43–46 injections). Individual case details are presented in Supplementary Table S3.

### 3.9. Safety

Across all 14 studies, no serious adverse events were reported. Only one study (Godse et al. [23]) reported a single case of injection site redness. Chaudhari et al. [22] reported transient wheals after injection in 4/30 patients in the AST group but none in the histaglobulin group. No systemic adverse reactions, anaphylaxis, or treatment discontinuation due to adverse effects were reported in any study. These observations should nonetheless be interpreted conservatively. No serious adverse events were identified within the available literature; however, the cumulative sample size (N = 754) is insufficient to exclude rare or long-term adverse effects, follow-up rarely exceeded 6 months, and most studies did not employ systematic, pre-specified adverse event ascertainment. With 754 exposed patients and no observed serious event, the upper 95% confidence limit for the true incidence of a serious adverse event is approximately 0.4%, so that events occurring less frequently than about 1 in 250 treated patients would not reliably have been detected.

### 3.10. Risk of Bias and Quality of Evidence

Risk of bias assessment revealed that the majority of studies were rated as “fair” quality using the NIH tools (Table 4). The primary limitations were absence of control groups in most studies (11/14 studies were single-arm), lack of blinding, small sample sizes, and potential for selection bias. Only two studies (Chaudhari et al. [22], Kumar S et al. [21]) employed randomization. The overall certainty of evidence was graded as “low” for chronic urticaria (due to study design limitations and heterogeneity) and “very low” for allergic rhinitis (due to absence of controlled trials and inconsistent outcome reporting) according to the GRADE framework (Table 5). Individual domain ratings for each study are shown in Supplementary Figure S2, and the overall distribution of risk of bias across domains is presented in Supplementary Figure S3.

### 3.11. Discussion

To our knowledge, this is the first meta-analysis evaluating the efficacy of histaglobulin in allergic diseases. The pooled analysis of six chronic urticaria studies demonstrated a large and statistically significant within-group effect (Hedges’ g = 2.82), indicating a substantial reduction in urticaria disease activity following histaglobulin treatment. This effect was robust across sensitivity analyses, remaining significant regardless of which study was excluded and across various assumed pre–post correlation values. It must be emphasized, however, that this estimate was obtained by pooling pre-treatment and post-treatment scores within the same cohorts rather than by comparing treated patients with an untreated, placebo, or active control group. The pooled Hedges’ g therefore reflects change over time rather than treatment efficacy in the causal sense. Part of the observed improvement may be attributable to regression to the mean, to spontaneous remission and the natural fluctuation of chronic urticaria, to placebo and non-specific care effects, and to concomitant therapies such as antihistamines or short courses of corticosteroids that were permitted in several of the included studies. None of these competing explanations could be quantified within the present design. Accordingly, the findings should be read as demonstrating an overall reduction in disease activity following histaglobulin treatment, rather than as definitive evidence of treatment effectiveness.

The magnitude of effect observed (g = 2.82) is notably large compared to other immunotherapeutic interventions for chronic urticaria. For context, omalizumab 300 mg demonstrated a UAS7 reduction of approximately 44% in the GLACIAL trial [32], while cyclosporine has shown approximately 65% improvement in responders [33]. In the present analysis, histaglobulin studies reported UAS reductions of 44–89%, suggesting comparable or potentially superior improvement, albeit derived from lower-quality evidence. This comparison is indirect and should be regarded as hypothesis-generating only, since the omalizumab and cyclosporine estimates derive from randomized placebo-controlled trials, in which the placebo response has been subtracted, whereas the histaglobulin estimates derive from uncontrolled before–after cohorts, in which it has not. It should be noted that the current EAACI/GA^2^LEN guideline [2] and BSACI guideline [4] recommend omalizumab as second-line and cyclosporine as third-line therapy for antihistamine-refractory chronic urticaria, yet neither guideline mentions histaglobulin. This omission likely reflects the lack of synthesized evidence rather than evidence of inefficacy, further underscoring the importance of the present review.

From an otorhinolaryngology perspective, the findings of this review carry important implications. Allergic rhinitis is one of the approved indications for histaglobulin, and the three included AR studies consistently demonstrated clinical improvement with response rates of 57–90%. The study by Verma et al. [29] is particularly noteworthy, reporting an 88% response rate with significant normalization of serum IgE (94%) and eosinophil counts (84%), suggesting not merely symptomatic relief but immunomodulatory effects relevant to the underlying pathophysiology of allergic rhinitis. These findings are consistent with the ARIA guideline’s emphasis [1] on treatments that address both symptoms and underlying inflammation. Narayana et al. [28], despite reporting a more modest response rate (59.6%), demonstrated that histaglobulin alone was as effective as histaglobulin combined with antihistamines, suggesting an independent immunomodulatory mechanism rather than a mere antihistamine-augmenting effect. Furthermore, the Russian experience reported by Gushchin et al. [34] demonstrated therapeutic effectiveness of histaglobin preparations in both allergic rhinitis and chronic urticaria, supporting the cross-disease efficacy observed in our review.

The mechanism of histaglobulin is particularly relevant to allergic rhinitis management. Unlike conventional antihistamines that merely block H1 receptors symptomatically, histaglobulin addresses multiple pathophysiological steps: inducing anti-histamine antibodies (histaminopexy), reducing IgE levels, and inhibiting eosinophil accumulation—the latter being a hallmark of allergic inflammation in the nasal mucosa [10,35]. Yoshii et al. [10] demonstrated in a mouse model that the histamine/γ-globulin complex selectively inhibits eosinophil but not neutrophil accumulation at inflammation sites, a finding with direct relevance to eosinophilic nasal inflammation in allergic rhinitis [35]. Durham et al. [35] demonstrated that messenger RNA for the type 2 cytokines interleukin (IL)-3, IL-4, and IL-5 is upregulated in the nasal mucosa of patients with allergic rhinitis after local allergen provocation, driving local eosinophil recruitment and activation. Type 2 immunity constitutes the core immunological programme of allergic rhinitis and of allergic disease generally. IL-4 and IL-13 drive immunoglobulin class switching to IgE, goblet cell metaplasia, and epithelial barrier dysfunction; IL-5 governs eosinophil differentiation, survival, and tissue recruitment; IL-9 supports mast cell expansion; and IL-3, together with IL-5 and granulocyte–macrophage colony-stimulating factor, sustains eosinophil and basophil maturation. These type 2 cytokines are produced not only by Th2 lymphocytes but also by group 2 innate lymphoid cells (ILC2s) activated by the epithelial alarmins thymic stromal lymphopoietin, IL-25, and IL-33, and their expression is further shaped by genetic and epigenetic regulation of the type 2 locus [36]. The selective eosinophil-inhibiting property of histaglobulin [10] therefore addresses a key pathological mechanism in allergic rhinitis. Additionally, Ayoub et al. demonstrated that histaglobulin inhibits nuclear factor-κB (NF-κB) nuclear translocation and downregulates tumour necrosis factor (TNF)-α and IL-6 [11], which are innate proinflammatory (non-type 2) cytokines; this indicates an action on the innate inflammatory compartment in addition to any effect on adaptive type 2 responses. The same group also reported modulation of the Th1/Th2 balance toward Th1 dominance in an ovalbumin allergy mouse model [12]. The Th1/Th2 paradigm, to which the available histaglobulin literature is largely confined, is nevertheless an incomplete description of allergic immunoregulation. Regulatory T cells (Tregs), particularly IL-10- and transforming growth factor-β-producing subsets, are now regarded as the pivotal mediators of allergen tolerance and of the durable clinical benefit of allergen-specific immunotherapy, and regulatory B cells together with Th17, Th9, and follicular helper T cells further shape the type 2 response [36,37]. Whether histaglobulin induces Treg expansion or IL-10-dependent tolerance, or instead suppresses type 2 cytokine production directly, has never been examined; this is arguably the most important mechanistic gap in the field and should be addressed in future translational work. These multi-level immunomodulatory effects distinguish histaglobulin from conventional antihistamines and position it as a disease-modifying rather than merely symptom-relieving intervention.

Compared to allergen-specific immunotherapy (AIT), which requires identification of specific causative allergens and carries the risk of anaphylaxis during dose escalation, histaglobulin offers a non-allergen-specific approach with a superior safety profile. This distinction is particularly relevant for patients with polysensitization or those in whom specific allergens cannot be identified—common clinical scenarios in otorhinolaryngology practice. Additionally, the relatively short treatment course (typically 8–10 weekly injections) compares favorably with the 3–5-year commitment required for conventional AIT. The Korean case series by Kim and Noh [31] and Noh [30] are noteworthy in demonstrating that longer treatment courses (12–46 weekly injections) using Histobulin™ can induce sustained remission lasting 10–52 months in chronic spontaneous urticaria, suggesting a potential disease-modifying effect beyond symptom suppression. This is consistent with the mechanism of enhanced histaminopexy [8], which may provide durable immunological tolerance rather than transient receptor blockade.

The cost-effectiveness of histaglobulin also merits consideration. At approximately USD 5–10 per injection in India and Korea, the total treatment cost for a standard 8-week course is substantially lower than omalizumab (approximately USD 500–1000 per injection) or even subcutaneous AIT. In resource-limited settings where allergic rhinitis imposes a significant quality-of-life burden, histaglobulin may represent a viable therapeutic option. The formulation stability and standardization of histaglobulin have been validated through modern analytical methods, including HPLC quantification of histamine content in immunoglobulin preparations [7], providing pharmaceutical quality assurance comparable to other biologic preparations.

A striking feature of the evidence base is its geographical concentration: all 14 included studies were conducted in Asia, 12 in India and 2 in the Republic of Korea, and no eligible study was identified from Europe, the Americas, Africa, or Oceania. This most probably reflects the regulatory and prescribing landscape rather than any ethnic or biological specificity. Histaglobulin is licensed and actively marketed in India, the Republic of Korea, Japan, and the Russian Federation, but it has never been approved in the United States or in most of the European Union, so investigators outside these markets have had neither access to the product nor an incentive to study it. Historical European and Russian experience with histaglobin preparations does exist [8,34], but it is published largely in Polish, Russian, German, and Japanese and was therefore excluded by our English-language restriction. Two consequences follow. First, geographical publication bias cannot be excluded: regionally circulating journals with limited international indexing may preferentially publish positive findings, and the funnel plot asymmetry described in Section 3.5 is compatible with this. Second, generalisability to non-Asian populations remains unproven, and replication in ethnically and geographically diverse cohorts is required before histaglobulin can be recommended more broadly.

It is equally informative to specify the allergic conditions for which our search retrieved no eligible clinical data. Although bronchial asthma is an approved indication for histaglobulin in several countries, we identified no English-language study evaluating histaglobulin in asthma as a primary indication; the only asthma-related data came from a mixed cohort in which patients with asthma and allergic rhinitis were analysed together for serum IgE [13]. Similarly, no eligible study was found in mastocytosis or mast cell activation syndrome, in the prevention of anaphylaxis or of systemic reactions during allergen-specific immunotherapy, in IgE-mediated food allergy, in chronic rhinosinusitis with nasal polyps, in allergic conjunctivitis, or in drug hypersensitivity. These are precisely the areas in which the therapeutic landscape has been reshaped by biologics: omalizumab is licensed for allergic asthma, chronic spontaneous urticaria, IgE-mediated food allergy, and chronic rhinosinusitis with nasal polyps, is used as an adjunct to allergen-specific immunotherapy and in mastocytosis-associated anaphylaxis, and has shown symptomatic benefit in allergic rhinitis in randomized trials and meta-analyses [32,38], while dupilumab, mepolizumab, benralizumab, and tezepelumab occupy overlapping type 2 indications. The complete absence of histaglobulin data across this spectrum is not evidence of ineffectiveness; it is a measure of how little the preparation has been examined by contemporary standards, and it defines an explicit research agenda.

These observations argue for a direct comparison between non-allergen-specific immunotherapy with histaglobulin and the two established alternatives, namely allergen-specific immunotherapy and anti-IgE or anti-type 2 biologic therapy. Such a comparison is clinically meaningful because the three approaches differ fundamentally in their prerequisites, cost, duration, and risk. Allergen-specific immunotherapy requires identification of a clinically relevant allergen, 3–5 years of treatment, and carries a small but real risk of systemic reactions; biologics require no allergen identification but cost two to three orders of magnitude more per injection; histaglobulin requires neither allergen identification nor substantial expenditure and appears well tolerated, but its evidence base is by far the weakest of the three. Head-to-head randomized trials—for example, histaglobulin versus subcutaneous or sublingual immunotherapy in monosensitized allergic rhinitis, and histaglobulin versus omalizumab in antihistamine-refractory chronic urticaria—using shared clinical outcomes, immunological endpoints (allergen-specific IgG4, Treg frequency, basophil activation), and formal cost-effectiveness analysis would determine whether non-specific immunotherapy has a defensible place in current treatment algorithms or should be regarded as a historical option.

The observed large effect sizes are supported by a plausible multi-level mechanistic framework. Histaglobulin appears to act at multiple points in the allergic cascade: (1) increasing histaminopexy at the effector level (step 4); (2) inhibiting mast cell degranulation (step 3); (3) decreasing allergen-specific IgE (step 1); and potentially (4) providing small-dose immunoglobulin effects analogous to intravenous immunoglobulin (IVIG), which may address autoimmune components of chronic urticaria [31]. The requirement for incubation of histamine with γ-globulin (1–2 h) to achieve optimal eosinophil inhibition [10] suggests that the complex itself, rather than its individual components, possesses the therapeutic activity—a finding consistent with the clinical observation that neither histamine nor γ-globulin alone produces therapeutic benefit.

Strengths of this review include: (1) being the first meta-analysis on histaglobulin; (2) comprehensive search across four databases; (3) inclusion of all allergic disease subtypes; and (4) multiple sensitivity analyses confirming the robustness of findings. However, several important limitations must be acknowledged. First, the high heterogeneity (I^2^ = 98.2%) reflects substantial differences in UAS scoring systems, treatment protocols, and study populations. Second, and most importantly, most included studies were single-arm prospective studies without control groups, so that the meta-analysis pooled within-cohort pre–post change rather than a between-group contrast. This precludes separation of any true treatment effect from the placebo effect—which can be substantial in urticaria (up to 20–30%)—and from spontaneous remission, the natural fluctuation of chronic urticaria, regression to the mean, and permitted concomitant medication. Third, the evidence for allergic rhinitis remains limited to three uncontrolled studies without standardized continuous outcome measures, permitting only narrative synthesis. Fourth, all included studies were conducted in Asia (12 in India and 2 in the Republic of Korea), which limits generalizability and raises the possibility of geographical publication bias. Fifth, the restriction to English-language publications may have excluded relevant Russian and Japanese studies. Sixth, the small number of studies in the meta-analysis (k = 6) limited the ability to assess publication bias formally and precluded formal subgroup analysis or meta-regression, so that the very high heterogeneity (I^2^ = 98.2%) remains only partially explained. Finally, although no serious adverse event was reported, the cumulative sample of 754 patients and the short duration of follow-up are insufficient to exclude rare or delayed harms. Despite these limitations, the consistency of positive findings across 14 independent studies [13,19,20,21,22,23,24,25,26,27,28,29,30,31], conducted by different research groups across two countries over nearly three decades, provides a degree of external validity that partially mitigates, but by no means removes, the internal validity concerns inherent to uncontrolled study designs.

Based on the findings of this review, several priority research directions emerge. First and foremost, well-designed randomized, double-blind, placebo-controlled trials of histaglobulin in allergic rhinitis are urgently needed, using standardized outcome measures such as the Total Nasal Symptom Score (TNSS) and Rhinoconjunctivitis Quality of Life Questionnaire (RQLQ). These trials should include objective nasal measures (nasal peak inspiratory flow, acoustic rhinometry) and biomarkers (nasal eosinophil counts, serum IgE) to establish both clinical efficacy and immunomodulatory mechanisms. Second, head-to-head comparisons with existing treatments (allergen-specific immunotherapy, omalizumab) would help position histaglobulin within treatment algorithms. Third, pharmacokinetic and pharmacodynamic studies examining the optimal dosing regimen, antibody kinetics, and duration of effect are needed. Fourth, studies in diverse populations beyond India and Korea would enhance generalizability. Notably, the Russian literature [34] and Japanese preclinical work [10] suggest broader international interest in histaglobulin, and multicenter international trials would be particularly valuable. Fifth, the promising case series data on remission induction in atopic dermatitis and chronic spontaneous urticaria [30,31] warrant investigation in larger controlled trials, given the significant unmet need in these conditions. Sixth, biomarker-guided patient selection (e.g., using autologous serum skin test status, baseline IgE levels, or histaminopexy capacity) could help identify optimal responders and move toward personalized immunotherapy. Seventh, mechanistic studies should move beyond the Th1/Th2 framework and determine whether histaglobulin induces regulatory T cell responses or IL-10-dependent tolerance, or suppresses type 2 cytokine production directly, using contemporary immunological readouts. Finally, because the present pooled estimate reflects within-group change only, a single adequately powered placebo-controlled trial would contribute more to the evidence base than any number of further uncontrolled cohorts.

## 4. Conclusions

This systematic review and meta-analysis provides the first quantitative synthesis of histaglobulin efficacy across allergic diseases. Meta-analysis of six chronic urticaria studies demonstrated a large pooled within-group effect size (Hedges’ g = 2.82; 95% CI 1.75–3.88). Available evidence therefore suggests that histaglobulin may reduce disease activity in chronic urticaria; however, confidence in the estimated effect remains limited, because the evidence derives predominantly from uncontrolled before–after studies with substantial heterogeneity (I^2^ = 98.2%) and reflects improvement following treatment rather than a controlled estimate of efficacy. For allergic rhinitis—one of its approved indications and a condition of primary interest to otorhinolaryngologists—preliminary uncontrolled evidence suggests that histaglobulin may provide benefit, with reported response rates of 57–90% and favourable immunomodulatory effects on IgE and eosinophils; these data are, however, limited and heterogeneous, and larger, well-designed controlled studies are needed to confirm effectiveness. No serious adverse events were identified within the available literature, but the cumulative sample size remains insufficient to exclude rare or long-term adverse effects. However, the low to very low certainty of evidence underscores the need for well-designed randomized controlled trials, particularly for allergic rhinitis. Given its favorable safety profile, low cost, ease of administration, and non-allergen-specific mechanism, histaglobulin merits rigorous re-evaluation as a potentially underutilized immunotherapeutic option; adequately powered placebo-controlled trials, together with direct comparisons against allergen-specific immunotherapy and biologics, are required before it can be positioned within evidence-based otorhinolaryngology and allergy practice.

## Figures and Tables

**Table 1:** Characteristics of included studies (*n* = 14).

Study	Country	Design	Disease	N *	Age (yr)	Treatment Protocol	Outcome Measure	Follow-Up
Rajesh 2016 [19]	India	Prospective	CU	51/29	37.2 ± 13.1	1 mL SC weekly × 8 wk	UAS (max 33/d)	24 wk
Khemani 2021 [20]	India	Prospective	CU	40/32	32.8	1 mL SC weekly × 8 wk	UAS7 (max 42/wk)	24 wk
Kumar S 2021 [21]	India	Comparative †	CU	48/34	30.0 ± 2.3	1 mL SC weekly × 6 wk	UAS	12 wk
Chaudhari 2019 [22]	India	RCT †	CU	30/30	31.3 ± 8.1	1 mL SC weekly × 8 wk	UAS (max 18)	24 wk
Godse 2019 [23]	India	PMS	CU	38/37	36.8 ± 10.2	1 mL SC × 3 doses	UAS7 (max 42/wk)	6 wk
Pavani 2025 [24]	India	Prospective	CU	28/28	38.2 ± 9.7	1 mL SC weekly × 8 wk	UAS	24 wk
Kaur 2024 [25]	India	Retrospective	CU	45/28	29.4	1 mL SC weekly × 8 wk	UAS7	—
Ravi 2024 [26]	India	Comparative †	CU	80/40 ‡	34	1 mL SC weekly × 8 wk	UAS	8 wk
Kumar YH 2016 [27]	India	Prospective	CU	200/110	30.2	1 mL SC weekly × 5 + 3,6 mo	Categorical	6 mo
Narayana 1997 [28]	India	Prospective §	AR	54/54	11–57	2 mL SC weekly × 10 + monthly × 3	Categorical	6 mo
Verma 2018 [29]	India	Prospective	AR	50/50	22	1 mL SC q4d × 8 + monthly × 3	Categorical + IgE	12 mo
Manohar 2012 [13]	India	Prospective	AR + Asthma	61/61	All ages	HG + drugs	Serum IgE	NR
Noh 2021 [30]	Korea	Case series	AD	4/4	15–20	2 mL SC weekly × 24–36 wk	SCORAD	Post-tx
Kim & Noh 2021 [31]	Korea	Case series	CSU	4/4	51–69	2 mL SC weekly until remission	USS	10–52 mo

CU, chronic urticaria; AR, allergic rhinitis; AD, atopic dermatitis; CSU, chronic spontaneous urticaria; SC, subcutaneous; PMS, post-marketing surveillance; HG, histaglobulin; NR, not reported; UAS, Urticaria Activity Score; IgE, immunoglobulin E. * Enrolled/analyzed. † Comparator: autologous serum therapy. ‡ Histaglobulin arms only. § Three arms: HG alone, HG + terfenadine, HG + cetirizine. AST, autologous serum therapy; RCT, randomized controlled trial; SCORAD, Scoring Atopic Dermatitis; USS, urticaria severity score; UAS7, weekly Urticaria Activity Score; d, day; wk, week; mo, month; yr, year.

**Table 2:** Meta-analysis of histaglobulin efficacy in chronic urticaria: pre–post UAS reduction (k = 6, N = 190).

Study	N	Pre-Treatment UAS (Mean ± SD)	Post-Treatment UAS (Mean ± SD)	Reduction (%)	Hedges’ g (95% CI)
Rajesh 2016 [19]	29	18.9 ± 6.30	3.7 ± 5.40	80.4	2.52 (2.16, 2.88)
Khemani 2021 [20]	32	12.6 ± 3.93	2.57 ± 2.21	81.0	3.07 (2.71, 3.43)
Kumar S 2021 [21] *	34	24.5 ± 2.65	11.8 ± 2.10	69.0	5.19 (4.86, 5.52)
Chaudhari 2019 [22]	30	12.6 ± 1.50	7.1 ± 2.77	43.7	2.40 (2.03, 2.78)
Godse 2019 [23]	37	15.8 ± 6.06	6.0 ± 6.22	62.0	1.56 (1.25, 1.88)
Pavani 2025 [24]	28	19.6 ± 8.40	3.9 ± 5.50	80.1	2.15 (1.77, 2.52)
**Pooled (random-effects)**	**190**	**—**	**—**	**—**	**2.82 (1.75, 3.88)**

Heterogeneity: I^2^ = 98.2%, τ^2^ = 1.75, Q = 277.4 (*p* < 0.001). Model: DerSimonian–Laird random-effects. Effect sizes are Hedges’ g (standardized mean difference with small-sample correction), computed as in Figure 2. Assumed pre–post correlation r = 0.5. * Kumar S 2021 [21] reported Mean ± 2SD; SD was derived by dividing by 2. CI, confidence interval; I^2^, I-squared heterogeneity statistic; k, number of studies; N, number of analyzed participants; Q, Cochran’s Q statistic; SD, standard deviation; UAS, Urticaria Activity Score; τ^2^, between-study variance.

**Table 3:** Sensitivity analyses of the pooled effect size in chronic urticaria.

	k	N	Hedges’ g (95% CI)	I^2^ (%)
**Primary analysis (all 6 studies, r = 0.5)**	**6**	**190**	**2.82 (1.75, 3.88)**	**98.2**
*Leave-one-out sensitivity analysis*				
Excluding Rajesh 2016 [19]	5	161	2.88 (1.58, 4.17)	98.5
Excluding Khemani 2021 [20]	5	158	2.77 (1.47, 4.06)	98.5
Excluding Kumar S 2021 [21]	5	156	2.34 (1.83, 2.85)	90.2
Excluding Chaudhari 2019 [22]	5	160	2.90 (1.62, 4.18)	98.5
Excluding Godse 2019 [23]	5	153	3.07 (1.93, 4.20)	98.0
Excluding Pavani 2025 [24]	5	162	2.95 (1.69, 4.21)	98.5
*Pre–post correlation sensitivity analysis*				
r = 0.3	6	190	2.82 (1.76, 3.87)	98.2
r = 0.5	6	190	2.82 (1.75, 3.88)	98.2
r = 0.7	6	190	2.82 (1.73, 3.91)	98.2
r = 0.8	6	190	2.82 (1.70, 3.93)	98.2

k, number of studies; N, total participants; CI, confidence interval; r, assumed pre–post correlation coefficient.

**Table 4:** Risk of bias assessment using NIH Quality Assessment Tools (*n* = 14).

Study	Q1	Q2	Q3	Q4	Q5	Q6	Q7	Q8	Q9	Overall
	*Objective*	*Eligibility*	*Represent.*	*Sample size*	*Interven. described*	*Outcomes defined*	*Blinding*	*Follow-up ≥80%*	*Statistics*	*Rating*
Rajesh 2016 [19]	Y	Y	CD	Y	Y	Y	N	N	Y	Fair
Khemani 2021 [20]	Y	Y	CD	N	Y	Y	N	Y	Y	Fair
Kumar S 2021 [21]	Y	Y	CD	Y	Y	Y	N	N	Y	Fair
Chaudhari 2019 [22]	Y	Y	Y	Y	Y	Y	P	Y	Y	Good
Godse 2019 [23]	Y	Y	CD	N	Y	Y	N	Y	Y	Fair
Pavani 2025 [24]	Y	Y	CD	N	Y	Y	N	Y	Y	Fair
Kaur 2024 [25]	Y	Y	CD	N	Y	Y	N	N	N	Poor
Ravi 2024 [26]	Y	Y	CD	N	Y	Y	N	Y	Y	Fair
Kumar YH 2016 [27]	Y	Y	Y	N	Y	N	N	N	Y	Fair
Narayana 1997 [28]	Y	Y	CD	N	Y	N	N	N	N	Poor
Verma 2018 [29]	Y	Y	CD	N	Y	Y	N	Y	Y	Fair
Manohar 2012 [13]	Y	Y	CD	N	Y	Y	N	Y	Y	Fair
Noh 2021 (AD) [30]	Y	Y	N	NA	Y	Y	N	Y	NA	Poor
Kim & Noh 2021 [31]	Y	Y	N	NA	Y	Y	N	Y	NA	Poor

Y, yes (low risk); N, no (high risk); P, partial; CD, cannot determine; NA, not applicable. Overall: Good, most criteria met with low risk of bias; Fair, some criteria met; Poor, significant limitations. NIH tools: Before–After (single-arm), Controlled Intervention (comparative/RCT), Case Series. NIH, National Institutes of Health; RCT, randomized controlled trial; AD, atopic dermatitis; Q1–Q9, items of the corresponding NIH quality assessment tool.

**Table 5:** GRADE evidence profile.

Outcome	Studies (N)	Risk of Bias	Inconsistency	Indirectness	Imprecision	Effect	Certainty
UAS reduction (CU)	6 (190)	Serious ^1^	Serious ^2^	Not serious	Not serious	g = 2.82 (1.75–3.88)	⊕⊕○○ LOW
Complete remission (CU)	5 (156)	Serious ^1^	Serious ^3^	Not serious	Serious ^4^	30–89%	⊕○○○ VERY LOW
Symptom improvement (AR)	3 (165)	Very serious ^5^	Not serious	Not serious	Serious ^4^	57–90%	⊕○○○ VERY LOW
Serum IgE reduction	2 (111)	Very serious ^5^	Not serious	Not serious	Serious ^4^	90–94% reduced	⊕○○○ VERY LOW
Safety (all diseases)	14 (754)	Serious ^1^	Not serious	Not serious	Serious ^6^	AE: 1/754 (0.13%)	⊕⊕○○ LOW

^1^ Most studies lacked control groups and blinding. ^2^ I^2^ = 98.2%; heterogeneity from different UAS scoring systems. ^3^ Wide range (30–89%) across studies. ^4^ Small total sample sizes. ^5^ No controlled trials; categorical outcomes only. ^6^ Rare events may not be captured in small studies. CU, chronic urticaria; AR, allergic rhinitis; UAS, Urticaria Activity Score; AE, adverse event; g, Hedges’ g; CI, confidence interval. GRADE, Grading of Recommendations, Assessment, Development, and Evaluations; I^2^, I-squared heterogeneity statistic; N, total number of participants; ⊕, filled circle denoting one level of certainty retained; ○, open circle denoting one level downgraded; ⊕⊕○○, low certainty; ⊕○○○, very low certainty.

## Data Availability

All data generated or analyzed during this study are included in this published article and its Supplementary Materials.

## Supplementary Materials

The following supporting information can be downloaded at: https://www.mdpi.com/article/10.3390/ijms27177753/s1.

## Abbreviations

The following abbreviations are used in this manuscript:

AR	Allergic rhinitis
AD	Atopic dermatitis
AIT	Allergen-specific immunotherapy
ASST	Autologous serum skin test
AST	Autologous serum therapy
CI	Confidence interval
CU	Chronic urticaria
CSU	Chronic spontaneous urticaria
GRADE	Grading of Recommendations, Assessment, Development, and Evaluations
IgE	Immunoglobulin E
IL	Interleukin
ILC2	Group 2 innate lymphoid cell
IVIG	Intravenous immunoglobulin
NIH	National Institutes of Health
PRISMA	Preferred Reporting Items for Systematic Reviews and Meta-Analyses
PROSPERO	International Prospective Register of Systematic Reviews
RQLQ	Rhinoconjunctivitis Quality of Life Questionnaire
SCORAD	Scoring Atopic Dermatitis
SD	Standard deviation
SMD	Standardized mean difference
SWiM	Synthesis Without Meta-analysis
Th1/Th2	T helper type 1/T helper type 2
TNF-α	Tumour necrosis factor-α
TNSS	Total Nasal Symptom Score
Treg	Regulatory T cell
UAS	Urticaria Activity Score
USS	Urticaria severity score
