# Supplementary material for: Efficacy of Histaglobulin in Allergic Diseases: A Systematic Review and Meta-Analysis with Implications for Allergic Rhinitis
Source: Int J Mol Sci. 2026 Aug 29;27(17):7753. doi: 10.3390/ijms27177753 (PMC13566228; doi:10.3390/ijms27177753)
Supplement: Supplementary file 1 [file ijms-27-07753-s001.zip › Histobulin_Supplementary_revised.pdf]

Supplementary Table S1. Full electronic search strategies for each database.

Database (platform)	Search query	Records
PubMed / MEDLINE (NCBI)	`(histaglobulin OR histobulin OR histaglobin OR "histamine immunoglobulin" OR "histamine gamma globulin" OR "histamine-fixed immunoglobulin" OR "immunoglobulin histamine complex" OR "histamine gamma-globulin" OR HISTOGLOB OR "histamine human gamma globulin") AND (rhinitis OR urticaria OR dermatitis OR asthma OR allergy OR allergic OR "allergic disease" OR "allergic diseases" OR immunotherapy)`	226
Embase (Elsevier; Emtree)	`(histaglobulin OR histobulin OR histaglobin OR 'histamine immunoglobulin' OR 'histamine gamma globulin' OR 'histamine-fixed immunoglobulin' OR histoglob) AND (rhinitis OR urticaria OR dermatitis OR asthma OR allergy OR allergic OR immunotherapy) AND [embase]/lim NOT ([medline]/lim)`	25
Web of Science (Clarivate; Core Collection)	`TS=(histaglobulin OR histobulin OR histaglobin OR "histamine immunoglobulin" OR "histamine gamma globulin" OR "histamine-fixed immunoglobulin" OR HISTOGLOB) AND TS=(rhinitis OR urticaria OR dermatitis OR asthma OR allergy OR allergic OR immunotherapy)`	148
Cochrane Library / CENTRAL (Wiley)	`histaglobulin OR histobulin OR histaglobin OR "histamine immunoglobulin" OR "histamine gamma globulin" OR HISTOGLOB`	17
**Total (before de-duplication)**		**416**

All databases were searched from inception to March 2026, with no date restriction; results were limited to English-language publications. In addition to the databases above, ClinicalTrials.gov, the WHO International Clinical Trials Registry Platform (ICTRP), Google Scholar, and the reference lists of included studies were searched for additional eligible records. Search syntax is presented as implemented on each platform; MeSH/Emtree mapping and field tags were adapted to each database. Record counts correspond to those reported in the study selection process (Section 3.1). Quotation marks are applied consistently to every multi-word search term: double quotation marks in PubMed, Web of Science and the Cochrane Library, and single quotation marks in Embase in accordance with Emtree platform syntax. CENTRAL, Cochrane Central Register of Controlled Trials; Emtree, Embase subject headings; ICTRP, International Clinical Trials Registry Platform; MeSH, Medical Subject Headings; TS, topic search field tag.

Supplementary Table S2. Detailed results of allergic rhinitis studies (n = 3)

	Narayana 1997 [28]	Verma 2018 [29]	Manohar 2012 [13]
**Study design**	Prospective, 3-arm	Prospective, single-arm	Prospective, single-arm
**Sample size**	54 (18 per arm)	50	61
**Population**	AR, 11–57 yr, 26M:28F	AR, 16–50 yr, refractory to conventional therapy	AR + Asthma, all ages, 29M:32F
**Dosing protocol**	2 mL SC weekly ×10 wk + monthly booster ×3 mo	1 mL SC: 4 inj q4d ×2 mo, monthly ×3 mo, booster at 6 mo	Histaglobulin + antihistamines + mast cell stabilizers + bronchodilators
**Outcome measures**	Clinical symptom grading (categorical: severe/ moderate/mild)	Symptom severity (categorical) + serum IgE + absolute eosinophil count	Serum IgE (pre vs. post treatment)
**Symptom results**	Moderate response: 7/18 (HG alone) 8/18 (HG + terfenadine) 2/18 (HG + cetirizine)	Nasal obstruction: 92%→12% Rhinorrhea: 84%→8% Sneezing: 84%→6% Itching: 84%→6%	Clinical improvement correlated with IgE reduction
**IgE change**	No significant change	94% normalized (47/50)	90.2% showed reduction (55/61 patients)
**Eosinophil change**	No significant change	84% normalized (44/50)	NR
**Overall response rate**	59.6% (HG alone) 61.7% (HG + terfenadine) 56.9% (HG + cetirizine)	88% (complete 46% + fair 42%)	90.2% (IgE-based)
**Recurrence**	2/13 at 6 months	6/50 at 12 months	NR
**Adverse events**	None reported	None reported	None reported

AR, allergic rhinitis; F, female; HG, histaglobulin; IgE, immunoglobulin E; inj, injections; M, male; mo, month; NR, not reported; q4d, every 4 days; SC, subcutaneous; wk, week; yr, year. No study included an untreated or placebo arm; response rates are within-group.

Supplementary Table S3. Individual case details from AD and CSU case series

Study	Case	Age/Sex	Disease	Duration	Severity before tx	No. of injections	Severity after tx	Improvement	Sustained remission	Follow-up
Noh 2021 [30]	1	19/F	AD	Infancy	SCORAD 42.5	36	SCORAD 0	100%	Yes	3 mo
	2	15/M	AD	Infancy	SCORAD 29	24	SCORAD 16.5	43%	No	Post-tx
	3	15/M	AD	Infancy	SCORAD 26.1	24	SCORAD 14.2	46%	No	Post-tx
	4	20/F	AD	Infancy	SCORAD 32.9	24	SCORAD 5	85%	No	Post-tx
Kim & Noh 2021 [31]	1	69/F	CSU	5 mo	USS 38	12	USS 0	100%	Yes (22 mo)	22 mo
	2	63/F	CSU	36 mo	USS 40	12	USS 0	100%	Yes (52 mo)	52 mo
	3	53/F	CSU	3 mo	USS 50	46	USS 0	100%	Yes (10 mo)	10 mo
	4	51/F	CSU	4 mo	USS 42	46	USS 0	100%	Yes (24 mo)	24 mo

AD, atopic dermatitis; CSU, chronic spontaneous urticaria; SCORAD, Scoring Atopic Dermatitis (max 103); USS, Urticaria Severity Score (Jariwala, max 93); tx, treatment; F, female; M, male; mo, month; SC, subcutaneous. These are uncontrolled case series; the reported improvements are within-patient changes following treatment.

Noh 2021: all patients also showed reduced frequency of upper respiratory infections during treatment. Kim & Noh 2021: Histobulin™ 2 mL SC weekly until clinical remission; all patients remained in sustained remission during follow-up.
